# Rapid-flow expulsion maneuver in subglottic secretion clearance to prevent ventilator-associated pneumonia: a randomized controlled study

**DOI:** 10.1186/s13613-021-00887-5

**Published:** 2021-06-24

**Authors:** Ying Li, Xue Yuan, Bing Sun, Hai-chao Li, Hui-wen Chu, Li Wang, Yu Zhao, Xiao Tang, Rui Wang, Xu-yan Li, Zhao-hui Tong, Chen Wang

**Affiliations:** 1 Department of Respiratory and Critical Care Medicine, Beijing Chao-Yang Hospital, Capital Medical University, No.8 Gongtinan Road, Beijing, 100020 China; 2 Beijing Institute of Respiratory Medicine, Beijing, China; 3 Beijing Key Laboratory of Respiratory and Pulmonary Circulation Disorders, Beijing, China; 4 Beijing Engineering Research Centre for Diagnosis and Treatment of Respiratory and Critical Care Medicine (Beijing Chao-Yang Hospital), Beijing, China; 5 Department of Pulmonary and Critical Care Medicine, Center of Respiratory Medicine, China-Japan Friendship Hospital, Beijing, China; 6 National Clinical Research Center for Respiratory Diseases, Beijing, China; 7 Chinese Academy of Medical Sciences and Peking Union Medical College, Beijing, China; 8 Department of Respiratory Medicine, Capital Medical University, Beijing, China

**Keywords:** Rapid-flow expulsion maneuver, Subglottic secretion drainage, Ventilator-associated pneumonia

## Abstract

**Background:**

Following endotracheal intubation, clearing secretions above the endotracheal tube cuff decreases the incidence of ventilator-associated pneumonia (VAP); therefore, subglottic secretion drainage (SSD) is widely advocated. Our group developed a novel technique to remove the subglottic secretions, the rapid-flow expulsion maneuver (RFEM). The objective of this study was to explore the effectiveness and safety of RFEM compared with SSD.

**Methods:**

This study was a single-center, prospective, randomized and controlled trial, conducted at Respiratory Intensive Care Unit (ICU) of Beijing Chao-Yang Hospital, a university-affiliated tertiary hospital. The primary outcome was the incidence of VAP, assessed for non-inferiority.

**Results:**

Patients with an endotracheal tube allowing drainage of subglottic secretions (*n* = 241) were randomly assigned to either the RFEM group (*n* = 120) or SSD group (*n* = 121). Eleven patients (9.17%) in the RFEM group and 13 (10.74%) in the SSD group developed VAP (difference, − 1.59; 95% confidence interval [CI] [− 9.20 6.03]), as the upper limit of 95% CI was not greater than the pre-defined non-inferiority limit (10%), RFEM was declared non-inferior to SSD. There were no statistically significant differences in the duration of mechanical ventilation, ICU mortality, or ICU length of stay and costs between groups. In terms of safety, no accidental extubation or maneuver-related barotrauma occurred in the RFEM group. The incidence of post-extubation laryngeal edema and reintubation was similar in both groups.

**Conclusions:**

RFEM is effective and safe, with non-inferiority compared to SSD in terms of the incidence of VAP. RFEM could be an alternative method in first-line treatment of respiratory ICU patients.

*Trial registration* This study has been registered on ClinicalTrials.gov (Registration Number: NCT02032849, https://clinicaltrials.gov/ct2/show/NCT02032849); registered on January 2014

**Supplementary Information:**

The online version contains supplementary material available at 10.1186/s13613-021-00887-5.

## Background

Establishment of an artificial airway is an important treatment approach in critically ill patients, which is commonly complicated by ventilator-associated pneumonia (VAP). Duration of mechanical ventilation, length and cost of time in intensive care unit (ICU), antibiotic treatment and patient mortality are significantly increased by VAP [1, 2]. The main cause of VAP is the accumulation of secretions in the gap between the glottis and the cuff after intubation, which cannot be cleared by coughing. This leads to the spread of pathogens in the lower respiratory tract [3].

Several studies have confirmed the effectiveness of subglottic secretion drainage (SSD) in reducing the incidence of VAP [4, 5]. The use of an endotracheal tube with a subglottic suctioning lumen has been recommended by several VAP prevention guidelines in several countries including the United States, Canada, and China [6–8]. However, there are still some limitations to this procedure. For example, expensive specialized tubes are required, and the procedure is often accompanied by complications, such as airway mucosal injury, and poor drainage [9, 10].

Our team has developed an innovative technique to remove subglottic secretions, named the rapid-flow expulsion maneuver (RFEM). It uses a manual resuscitator to generate rapid-flow expulsion which can clear the subglottic secretions efficiently. It has been evaluated in in vitro and in vivo pre-trial investigations [11] and is patented and applied widely in more than 50 ICUs in China since the 1990s. RFEM has been shown to be safe, and cost-effective [12, 13]. Nevertheless, there was still a lack of evidence from large-scale randomized controlled trials (RCT) on the effectiveness of RFEM in preventing VAP. The technique RFEM has not been more widely evaluated or used in other countries around the world.

To obtain further evidence-based support for the wider use of RFEM, we performed this trial to explore the efficacy and safety of RFEM in preventing VAP compared with standard SSD.

## Methods

### Subjects

Patients intubated for less than 24 h in the respiratory ICU and aged 18 years or older were eligible for this trial if they had an estimated survival time > 2 weeks. Patients were excluded if they had: ventilation parameters with positive end-expiratory pressure (PEEP) > 10 cmH_2_O or fraction of inspired oxygen (FiO_2_) > 0.8; hemodynamic instability; history of severe pulmonary bullae with pneumothorax; positive cuff leak test, which means patients with upper airway obstruction, it is difficult to push the secretion up to the oropharynx [14, 15] or had been included in other clinical studies. During the study, patients that were withdrawn from mechanical ventilation after less than 72 h, or those whom refused treatment were also excluded.

### Trial design and randomization

This was a prospective, single-center, randomized, clinical control trial conducted in the Respiratory ICU at Beijing Chao-Yang Hospital, Capital Medical University (ClinicalTrials.gov, NCT02032849). This study was approved by the Ethics Committee of Beijing Chao-Yang Hospital (2014-KE-106) and informed consent was obtained from the patients or their surrogates.

Randomization was performed using random numbers generated by the random number generator in the SPSS 23.0 statistical software (IBM Corp., Armonk, NY, USA). The enrolled patients (*n* = 241) were randomly assigned to either the RFEM group (*n* = 120) or the SSD group (*n* = 121). Allocation concealment was conducted using sequentially numbered opaque sealed envelopes. This was an unblinded trial because the physicians were aware of the treatment assigned to every participant. However, during the entire study period, the endpoint judgement and the statisticians were blinded.

### Procedures

All patients enrolled in this study underwent endotracheal intubation along with a subglottic suctioning catheter (TaperGuard™ Evac Oral Tracheal Tube; Medtronic, USA). Clearance of subglottic secretions was performed every 6 h, and the secretion amounts were recorded.

#### Rapid-flow expulsion maneuver (RFEM)

A manual resuscitator was attached to endotracheal tube and the cuff deflated during the initiation of exhalation, the rapid flow produced by the manual resuscitator passing the space around the deflated cuff was used to remove subglottic secretions to the oropharynx. The operational procedure is completed by two operators (respiratory therapists or ICU nurses), described in Additional file 1: S1, and Additional file 2: Video S1 showed how RFEM works.

#### Subglottic secretion drainage (SSD)

A pressure of − 100 mmHg with a 15-s duration was applied through a subglottic secretion drainage catheter connected to the sputum collector to carefully suction oral and tracheal secretions while subjects were placed in a semi-recumbent position [11]. If the catheter became blocked, 5 ml of normal saline was instilled through the drainage lumen to maintain its patency [16].

### Data collection, quality control and VAP prevention-bundle

After informed consent was obtained from the study patients or surrogates, baseline data were recorded: age, sex, Acute Physiology and Chronic Health Evaluation (APACHE) II score, Sequential Organ Failure Assessment (SOFA) score at ICU admission, comorbidities, causes of tracheal intubation and laboratory examinations. Ventilator parameters were also recorded at randomization.

Daily data for a VAP-monitoring form were recorded for each enrolled patient and checked by five respiratory therapists. The diagnosis of VAP was initially made according to the VAP diagnostic criteria (Additional file 1: S2) by two blindly assigned ICU physicians. If the results were inconsistent, a microbiologist would participate to establish the diagnosis. Clinical data were recorded on paper case record forms then double-entered into an electronic database and validated by the trial staff.

Other measures were taken to prevent VAP in the two groups, including raising the head of the bed, oral care using chlorhexidine, rational use of sedative and analgesic drugs, maintenance of cuff pressure within 25–30 cmH_2_O, replacement of ventilator tubes only when visible stains or failure occurred, early limb rehabilitation exercise, and daily evaluation of extubation.

### Endpoints

The incidence of VAP was the primary endpoint of the study. Patients enrolled in the study were followed-up prospectively for the occurrence of VAP until they received a tracheotomy, were successfully weaned from mechanical ventilation, discharged from the hospital, or died. The per protocol population contains patients who had PEEP below 10 cmH2O or FiO_2_ below 0.8 at study randomization. These patients were included in the intention-to-treat analysis.

The secondary endpoints included, mechanical ventilation duration, time from intubation to VAP, length of and cost of ICU stay, and mortality while in ICU. The daily volume of subglottic secretions cleared and the need for tracheotomy and reintubation were also recorded.

The safety of RFEM was assessed by recording episodes of pneumothorax, unplanned extubation and changes in vital signs during the maneuver process. Incidence of the post-extubation laryngeal dyspnea in both groups was also evaluated as a safety factor.

### Statistical methods

#### Sample size calculation

The primary endpoint was evaluated using a non-inferiority analysis. Sample sizes of 120 participants per group achieve 80% power to detect a non-inferiority margin difference between the group proportions of 0.10, with a one-sided test significance level of 0.05, and a loss to follow-up rate of 10%. Based on the incidence rates of VAP in patients requiring mechanical ventilation in our ICU prior to this study and the results of previous studies [17–19], the SSD group proportion is 15%, and the RFEM group proportion is 16.7%.

SPSS 23.0 software (IBM Corp., Armonk, NY, USA) was used for statistical analysis. The level of significance for all statistical tests was 0.05 (two-tailed). The measurement data were presented as means ± SD (standard deviations) or medians and quartile distribution (skewed distribution). Differences between groups were analyzed using the analysis of variance or nonparametric test (skewed distribution). Count data were presented as frequencies and percentages, and differences between groups were tested using the *χ*^2^ test or Fisher’s exact test. VAP-free survival curves in the two groups were displayed graphically according to the Kaplan–Meier method and analyzed using the log-rank test. Univariate and multivariate logistic regression was used to analyze the risk factors for the prevalence of VAP.

## Results

### Patient characteristics

**Fig. 1:**
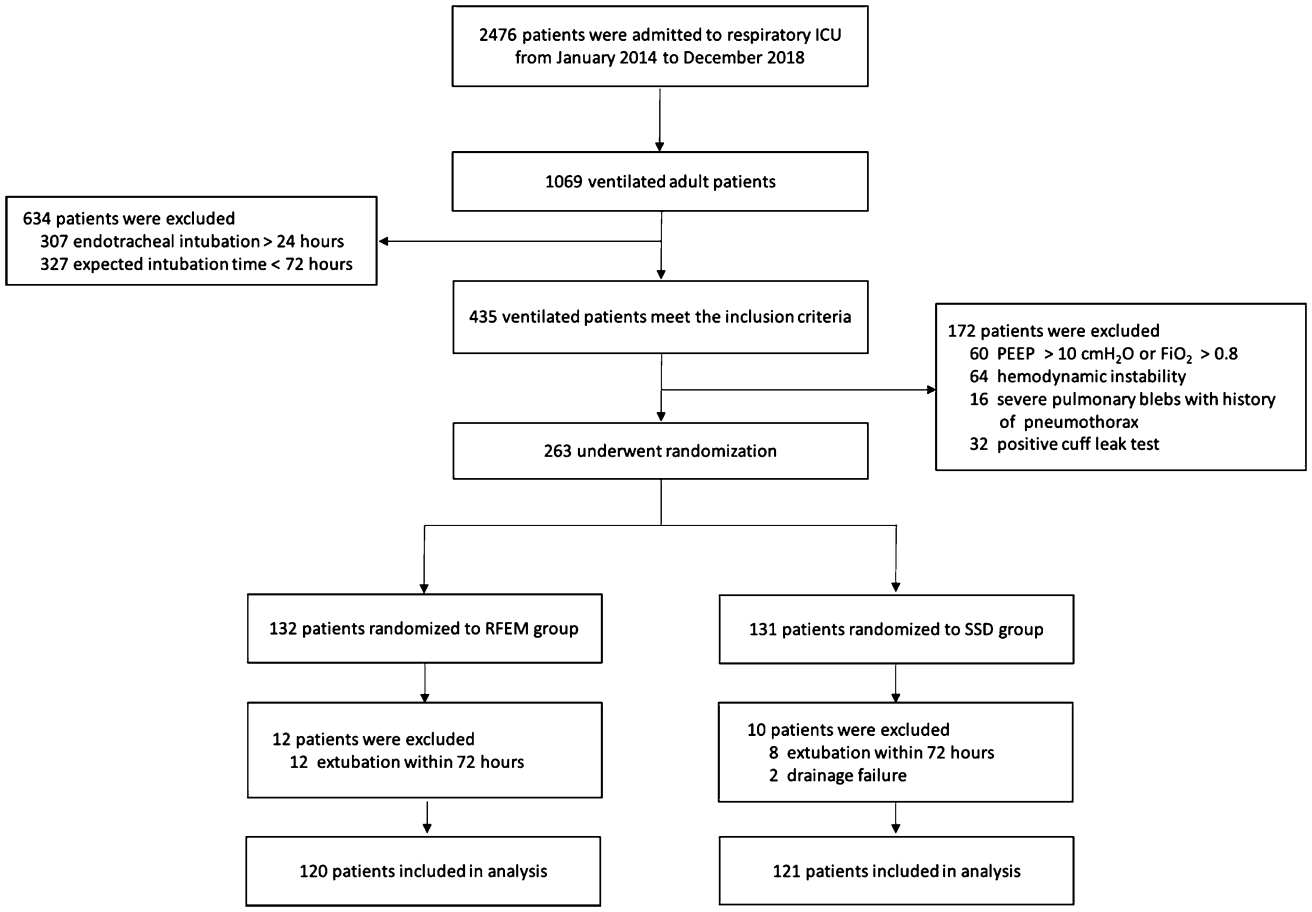
Flowchart of patients admitted to the respiratory intensive care unit (RICU) between January 2014 and December 2018

Figure 1 shows the flowchart of patients admitted to the respiratory intensive care unit (RICU) between January 2014 and December 2018. 1069 adult patients with mechanical ventilation were admitted, 806 of whom were excluded according to the selection criteria. Finally, 120 patients were included in the RFEM group and 121 patients were included in the SSD group.

There were no statistically significant differences between the two groups on study entry in a variety of factors including demographic data, comorbidities, Apache II or SOFA scores, hemodynamic status and laboratory examinations (Table 1, Additional file 1: Table S1). Table 1 lists the causes of tracheal intubation. The main cause for the two groups was respiratory failure (69.17% of the RFEM group and 75.21% of the SSD group) with no significant difference between the groups. The main causes of respiratory failure were pneumonia and exacerbation of chronic obstructive pulmonary disease (Additional file 1: Table S2). There were no statistically significant differences in arterial blood gas analysis, respiratory system compliance, or ventilator parameters, including PEEP, tidal volume, and plateau pressure at the time of enrollment (Table 2).

**Table 1:** Characteristics of study patients at randomization

Patient characteristics	Total (*n* = 241)	RFEM (*n* = 120)	SSD (*n* = 121)	*p*
Age, years	61 ± 17	63 ± 16	58 ± 17	0.074
Gender male, *n* (%)	155 (64.32)	76 (63.33)	79 (65.29)	0.751
Apache II score	16 ± 6	16 ± 7	16 ± 6	0.865
SOFA score	7 ± 4	6 ± 3	7 ± 3	0.109
Comorbidities, *n* (%)				
Diabetes	35 (14.52)	22 (18.33)	13 (10.74)	0.095
Chronic respiratory diseases	16 (6.64)	11 (9.17)	5 (4.13)	0.117
Cardiovascular diseases	117 (48.55)	64 (53.33)	53 (43.80)	0.139
Chronic renal insufficiency	23 (9.54)	13 (10.83)	10 (8.26)	0.497
Solid tumors	24 (9.96)	12 (10.00)	12 (9.92)	0.983
Hematological neoplasms	16 (6.64)	7 (5.83)	9 (7.44)	0.617
Immunosuppressive therapy	43 (17.84)	20 (16.67)	23 (19.01)	0.635
Causes of tracheal intubation, *n* (%)				
Respiratory failure	174 (72.20)	83 (69.17)	91 (75.21)	0.295
Consciousness disorder	41 (17.01)	21 (17.50)	20 (16.53)	0.841
Shock	7 (2.90)	4 (3.33)	3 (2.48)	0.722
Heart failure	7 (2.90)	4 (3.33)	3 (2.48)	0.722
Other reasons	12 (4.98)	7 (5.83)	5 (4.13)	0.544

*APACHE* Acute Physiology and Chronic Health Evaluation, *SOFA* Sequential Organ Failure Assessment

**Table 2:** Ventilation function of patients at randomization

Patient characteristics	Total (*n* = 241)	RFEM (*n* = 120)	SSD (*n* = 121)	*p*
Arterial blood gas analysis				
pH	7.38 ± 0.10	7.38 ± 0.10	7.38 ± 0.10	0.898
PaO_2_ (mmHg)	76.6 ± 29.9	78 ± 34	75 ± 25	0.357
PaCO_2_ (mmHg)	49.1 ± 22.3	50 ± 24	48 ± 20	0.335
PaO_2_:FiO_2_ (mmHg)	152.7 ± 87.3	163 ± 84	147 ± 90	0.199
Ventilation parameters				
PEEP (mmH_2_O)	10(8–10)	10(8–10)	10(9–10)	0.547
Tidal volume (ml/PBM)	6.9 ± 2.6	7.2 ± 2.9	6.7 ± 2.3	0.354
Respiratory rate (beats/min)	30 ± 9	29 ± 8	31 ± 9	0.280
Plateau pressure (cmH_2_O)	25 ± 4	24 ± 4	26 ± 3	0.085
Static compliance (ml/cmH_2_O)	31.2 ± 14.4	34.4 ± 16.2	28.8 ± 12.6	0.124

*PaO*_*2*_ partial pressure of arterial oxygen, *PaCO*_*2*_ partial pressure of arterial carbon dioxide, *PaO*_*2*_*:FiO*_*2*_ the ratio of the partial pressure of arterial oxygen to the fraction of inspired oxygen, *PEEP* positive end-expiratory pressure, *PBM* predicted body weight

Additional file 1: Table S3 compares the risk factors for the development of VAP in both groups. There were no significant differences between the two groups in predisposing conditions.

### Primary endpoint

**Fig. 2:**
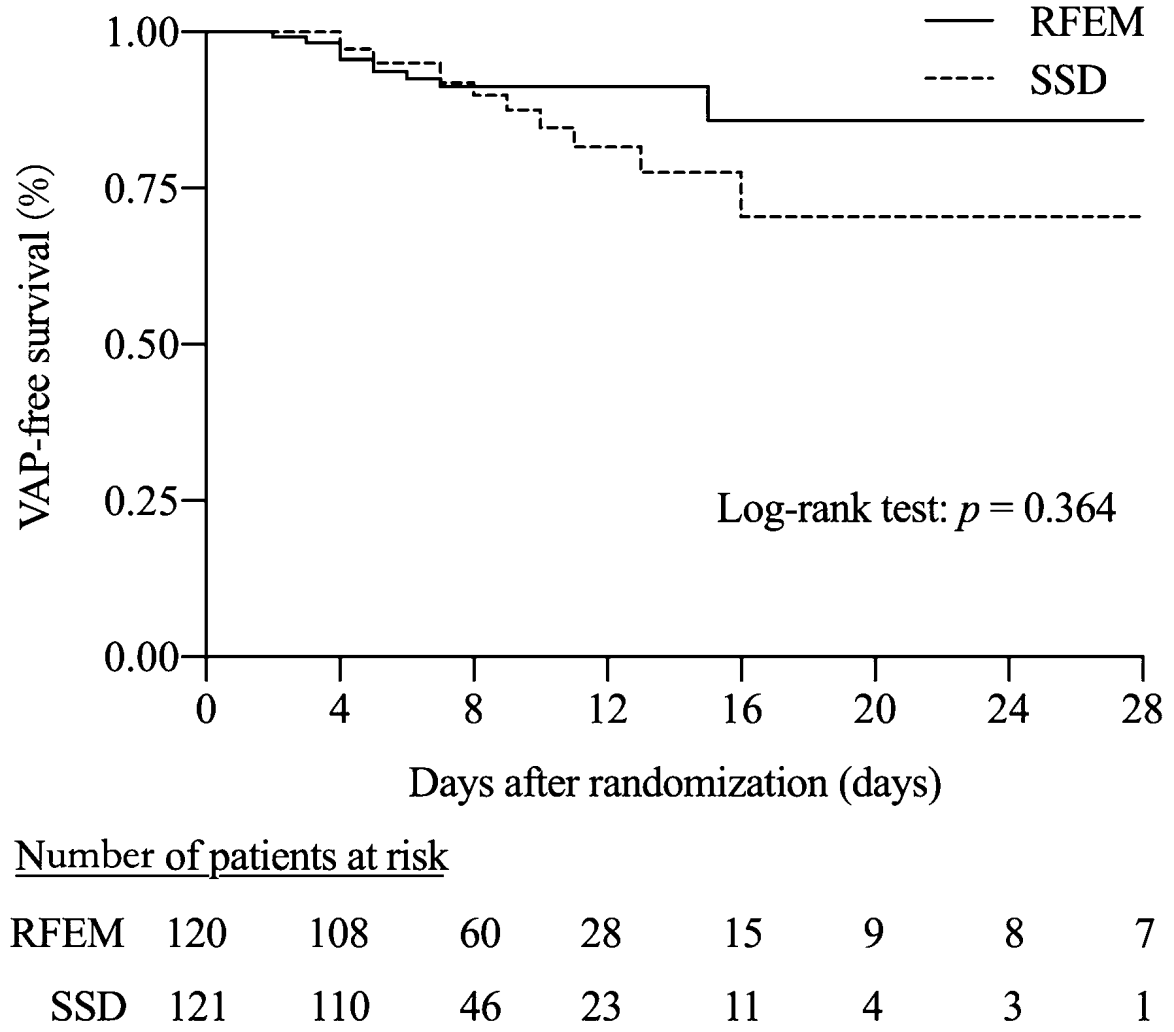
Kaplan–Meier curves of cumulative rates of patients remaining free of ventilator-associated pneumonia in two groups

In the analysis of the intention-to-treat population, the primary composite endpoint occurred in 11 (9.17%) patients in the RFEM group and in 13 (10.74%) patients in the SSD group, with an absolute risk difference of − 1.59% and a one-sided upper 95% confidence limit of 6.03% (*p* = 0.683 for non-inferiority, Table 3). The cumulative rates of patients remaining VAP-free in the two groups using the Kaplan–Meier curve showed that the rate of VAP-free patients in the RFEM group was numerically higher than that of the SSD group but without a statistically significant difference (log rank test, *p* = 0.364) (Fig. 2).

**Table 3:** Outcomes

	RFEM (*n* = 120)	SSD (*n* = 121)	Risk difference (95%CI)	*p*
Primary endpoint				
VAP, *n* (%)	11 (9.17)	13 (10.74)	− 1.59 (− 9.20, 6.03)	0.683
Secondary endpoints				
Duration of mechanical ventilation, days	8 (5–11)	7 (4–11)	1 (0, 2)	0.141
Time from intubation to VAP, days	7.25 ± 7.94	7.92 ± 3.77	− 0.67 (− 6.03, 4.69)	0.793
ICU length of stay, days	16 (10–25)	16 (9–26)	1 (− 2, 3)	0.643
ICU mortality, *n* (%)	36 (30.00)	46 (38.02)	− 8.08 (− 20.7, 3.91)	0.188
ICU expenses, thousand dollars	12.0 (7.88–19.5)	13.3 (7.69–25.2)	0.87 (− 3.28, 15.1)	0.465
Volume of subglottic secretions, ml/day	9.67 (6.78–13.18)	6.00 (2.10–10.76)	3.15 (1.42, 4.68)	< 0.001
Tracheotomy, *n* (%)	24 (20.00)	23 (19.01)	1 (− 9.08, 11.08)	0.846
Reintubation, *n* (%)	12 (10.00)	11 (9.09)	0.91 (− 6.56, 8.4)	0.810
Antibiotic days during ICU stay (%)	14 (10–20)	15 (8–20)	1 (− 2, 2)	0.601
Safety assessment, *n* (%)				
Barotrauma	6 (5.00)	10 (8.26)	− 3.29 (− 9.61, 3.03)	0.309
Pneumothorax	5 (83.33)	6 (60.00)	23.33 (− 19.22, 65.89)	0.330
Mediastinal emphysema	1 (16.67)	2 (20.00)	− 3.33 (− 42.11, 35.44)	0.869
Subcutaneous emphysema	1 (16.67)	4 (40.00)	− 23.33 (− 65.89, 19.22)	0.330
Post-extubation laryngeal edema	7 (5.83)	4 (3.31)	2.55 (− 2.76, 7.86)	0.347

Qualitative indicators are expressed by the number of cases (percentage), and 95% CI is calculated by normal approximation method. If the quantitative index obeys normal distribution, it is expressed by mean ± standard deviation, and 95% CI is calculated by *t*-test method; if it does not obey normal distribution, it is expressed by median (upper quartile—lower quartile), and 95% CI is calculated by Hodges–Lehmann estimation method

### Secondary endpoints

The time from endotracheal intubation to a VAP diagnosis was also similar between the two groups (7.25 ± 7.94 days, 7.92 ± 3.77 days, respectively *p* = 0.793). There was no significant difference in the duration of mechanical ventilation, ICU mortality, the length of ICU stay, or ICU cost between the two groups. ICU mortality was 30.00% in the RFEM group and 38.02% in the SSD group (*p* = 0.188) (Table 3).

The multivariate logistic regression analysis of risk factors associated with VAP showed that only duration of mechanical ventilation significantly increased the risk of VAP (OR = 1.047, 95%CI 1.008–1.087, *p* = 0.019). Ventilation duration was 13.3 ± 7.5 days in patients developing VAP and 8.9 ± 7.8 days in the others (*p* = 0.010).

Reintubation was required in 12 (10.00%) patients in the RFEM group and 11 (9.09%) patients in the SSD group without apparent clinical consequences. There was no significant difference in the incidence of pneumothorax, mediastinal emphysema, or subcutaneous emphysema between the two groups (Table 3).

SSD was performed at a median of 24 times (16–48 times) per patient and RFEM 32 times (20–48 times) per patient. The median daily volume of subglottic secretions cleared was 9.67 ml (6.78–13.18 ml) in the RFEM group, significantly higher than that of the SSD group (6.00 ml, 2.10–10.76 ml), *p* < 0.001.

### Diagnosis of VAP-associated microorganisms

There was also no significant difference in the etiological distribution between the two groups (Additional file 1: Table S4). Most of the pathogens responsible for VAP in the two groups were Gram-negative bacilli with the exception of *Streptococcus constellatus* in one patient of the RFEM group. *Acinetobacter baumannii* and *Pseudomonas aeruginosa* were the most commonly detected pathogens.

### Safety

In terms of safety, no accidental extubation or maneuver-related barotrauma occurred in the RFEM group. Changes in vital signs during the process of RFEM were recorded (Additional file 1: Table S5). The heart rate, blood pressure and respiratory rate were significantly increased during the maneuver process of RFEM. However, during the study, there were only 0.36% (14/3860) episodes of a delayed RFEM owing to abnormal vital signs. Post-extubation laryngeal edema occurred in 7 (5.83%) patients in the RFEM group and 4 (3.31%) in the SSD group (*p* = 0.347).

## Discussion

To the best of our knowledge, this is the first RCT to investigate the efficacy and safety of RFEM in preventing VAP compared with SSD. The major finding of our study is that there was no significant difference in the incidence of VAP between the two groups. RFEM can avoid the limitations of SSD, and we have verified RFEM to be a safe procedure without severe complications.

Several RCTs and meta-analyses show that SSD can significantly reduce the incidence of VAP [4, 5, 18–23]. In this study, the incidence of VAP in the SSD group was 10.83%, which was consistent with previous studies [19, 20]. Jason Powell’s study showed that, especially in critically ill patients, intubation and mechanical ventilation can cause an inflammatory subglottic environment where mucin hyper-secretion and enhanced viscosity is connected with neutrophil infiltration, impairment of neutrophil function, neutrophil elastase release, and enriched VAP-causing pathogens [3]. Enhancement of subglottic mucus removal and/or disruption could be considered a logical target for improved VAP prevention [3]. Other measures have been taken to prevent VAP, including elevating the head of the bed, daily oral hygiene, reducing the use of sedatives and strengthening cuff management [24–27]. The lower incidence of VAP in the SSD group in our results (10.8%) compared with the predicted incidence of VAP in the sample size calculation (30%) may be due to strict VAP bundles implementation and the fact that the diagnostic criteria for VAP remains a matter of debate [19]. Our study applied a particularly stringent diagnostic criterion which required specific microbiological vigilance (Additional file 1: Appendix S2).

Multiple studies have found that half of patients have a conventional tracheal tube established prior to ICU admission [19, 28–30], which limits the application of SSD. Furthermore, the price of the SSD tube is higher than a conventional tracheal tube. However, some studies have shown that the SSD method is more cost-effective for patients who are on mechanical ventilation > 48 h [31]; but the expected duration of intubation cannot be predicted at the beginning of treatment. In regards to the SSD lumen, the larger outer diameter of the catheter increases the risk of laryngeal injury [22]. Additionally, SSD may cause damage to the tracheal mucosa owing to the focus of negative pressure on the small amount of oropharyngeal secretion gathered above the balloon [9, 10, 32]. An in vitro study indicated that the SSD drainage effect is significantly reduced when the secretion above the cuff balloon was less than 4 ml [33]. Furthermore, the thinner diameter of the drainage tube can result in blockage by thick secretions.

Patients randomized to RFEM had a statistically similar incidence of VAP as patients in the SSD group. However, the RFEM does not require the SSD catheter or other special equipment and is not affected by the quantity and viscosity of the subglottic secretions. Therefore, the amount of daily subglottic secretions removed was greater in the RFEM group. In our study, we found patient heart rate, blood pressure and respiratory rate were significantly increased during the RFEM process. The process of sputum suction could partly explain these increases, as most patients returned to normal after a few minutes. No unplanned extubation or maneuver-related barotrauma occurred, and all conscious patients tolerated the procedure.

The technique RFEM applied widely in more than 50 ICUs in China, however, has not been more widely evaluated or used in other countries around the world nor it has been discussed as a potential strategy to prevent VAP in the different international recommendations or guidelines (due to the lack of evidence certainly). This study is the first randomized controlled study to investigate the efficacy and safety of RFEM in preventing VAP compared with SSD. Due to participation of a single center with relatively small sample size, the effectiveness and safety of RFEM appears promising, but there are far too little data at present to be able to make a statement with high confidence. We have also started to conduct follow-up research and try to cooperate with other centers in an attempt to include a larger sample size for verification and promotion.

The RFEM should be an alternative method for hospitals where the SSD catheter has not yet been popularized or for patients without the availability of subglottic suctioning catheters. It is worth noting, however, that RFEM has limitations under certain conditions. For patients requiring high PEEP support (e.g., PEEP > 10 cm H_2_O), the rapid-flow expulsion might cause the loss of PEEP and the collapse of alveoli when disconnecting patients from the ventilator. Furthermore, it is difficult to push the secretion up to the oropharynx in patients with an upper airway obstruction [14]. Otherwise, patients are supposed to lie in the supine position as much as possible to guarantee the most effective drainage [11]. Additionally, the cooperation of two trained medical staff is required at each time, these features limit the dissemination and appropriation of the technique by the ICU healthcare workers worldwide.

There are several limitations to our study. First, it was a single-center study with relatively small sample size, which was lacking a control group who received neither RFEM nor SSD, and the main cause of admission was respiratory infection. The small number of patients make it difficult to be confident of the relative safety of RFEM vs SSD. Large multi-center RCTs should be conducted to validate these findings and confirm the cost-effectiveness of RFEM. Second, the ICU expenses calculated in our study did not include the cost of human resource management. Lastly, the two operating procedures, RFEM and SSD, were visually distinguishable, thus the study could not be blinded to physicians and nurses. However, patients were randomized with similar baseline characteristics, and microbiologists blinded to the randomization used strict quantitative microbiological criteria to confirm VAP.

## Conclusions

For the clearance of subglottic secretions and prevention of VAP, RFEM has a non-inferior efficacy and safety to SSD, and therefore, may serve as an alternative method for SSD. Given the size and center limitations of the study, it would suggest much more cautious language regarding the safety and effectiveness of RFEM. It appears promising, but there are far too little data at present to be able to claim this with confidence. Large multi-center RCTs should be conducted to validate these findings and confirm the cost-effectiveness of RFEM.

## Supplementary Information

**Additional file 1: Appendix S1.** The standard operating procedure for the rapid-flow expulsion maneuver (RFEM). **Appendix S2.** The definition of VAP and VAP prevention-bundle. **Table S1.** Hemodynamic status and Laboratory examinations of study patients at randomization. **Table S2.** Tracheal intubation due to respiratory failure. **Table S3.** Risk factors for VAP during the study period. **Table S4.** Microorganisms diagnosis of VAP in patients. **Table S5.** Changes of vital signs during process of rapid-flow expulsion maneuver.

**Additional file 2: Video S1.** Rapid-flow expulsion maneuver.

## Data Availability

The authors confirm that all data generated or analyzed during this study are included in this published article and its additional information files.

## Abbreviations

APACHE II: Acute Physiology and Chronic Health Evaluation II
ICU: Intensive care unit
PEEP: Positive end-expiratory pressure
RCT: Randomized controlled trials
RFEM: Rapid-flow expulsion maneuver
SOFA: Sequential Organ Failure Assessment
SSD: Subglottic secretion drainage
VAP: Ventilator-associated pneumonia
