# Supplementary material for: Rapid-flow expulsion maneuver in subglottic secretion clearance to prevent ventilator-associated pneumonia: a randomized controlled study
Source: Ann Intensive Care. 2021 Jun 24;11:98. doi: 10.1186/s13613-021-00887-5 (PMC8222955; doi:10.1186/s13613-021-00887-5)
Supplement: Supplementary file 1 — Additional file 1: Appendix S1. The standard operating procedure for the rapid-flow expulsion maneuver (RFEM). Appendix S2. The definition of VAP and VAP prevention-bundle. Table S1. Hemodynamic status and Laboratory examinations of study patients at randomization. Table S2. Tracheal intubation due to respiratory failure. Table S3. Risk factors for VAP during the study period. Table S4. Microorganisms diagnosis of VAP in patients. Table S5. Changes of vital signs during process of rapid-flow expulsion maneuver. [file 13613_2021_887_MOESM1_ESM.doc]

**Rapid-Flow Expulsion Maneuver in Subglottic Secretion Clearance to Prevent Ventilator-Associated Pneumonia: A Randomized Controlled Study**

Additional file S1: The standard operating procedure for the rapid-flow expulsion maneuver (RFEM).

The standard operating procedure for the rapid-flow expulsion maneuver (RFEM) has been described in detail in our previous study^1^ .When vital signs are stable, stop nasal feeding for 30 minutes and withdraw gastrointestinal retention. Assist the patient to the supine or Trendelenburg position, and fully suction the secretions from the trachea, mouth and nasal cavity.

After connecting the breathing bag to the oxygen cylinder, adjust the oxygen flow rate to >10 L/min to keep the oxygen bag inflated, and deliver steady breaths via the resuscitation bag. At the beginning of expiration, the operator delivers high oxygen flow against the subject’s exhalation flow. At the same time, the assistant deflates the cuff, and the mixed flows are forced to expel through the surrounding space of the deflated cuff. The subglottic secretions will be brought out by the flow to the mouth. Repeat the procedure 2-3 times as appropriate until the secretions are completely removed.

During the maneuver, the clinicians monitor subject’s vital signs, SpO2, heart rhythm, and subjective complaints. The maneuver should be terminated if any of the following occur: (1) SpO2 ≤90%; (2) heart rate increased by 20%; (3) patient discomfort; or (4) new-onset irregular heart rhythm.

Quality control of RFEM：To verify the effectiveness of this method, we created a model in which the tracheal intubation was placed in a disposable ventilator tube (simulated airway). The intubation cuff balloon was inflated, and a colored liquid was injected over the balloon to simulate the subglottic secretion. The other end of the ventilator tube was connected to the simulated lung. Through this model, an in vitro study supposed that the median (interquartile range) clearance efficiencies of the first maneuver was 39.6% (18.1-65.4%), and visibly expelled in 86.1% of cases (93 of 108) (*p* <0.001)^1^. Respiratory therapists use this patented model for training to obtain proficiency in in vitro procedures prior to being involved in clinical work so there are no retentate leaks and ineffective operations.

**References:**

1. Li J, Zong YJ, Zhou Q, Dai H, Wang C. Evaluation of the Safety and Effectiveness of the Rapid Flow Expulsion Maneuver to Clear Subglottic Secretions in Vitro and in Vivo. Respir Care 2017; 62:1007-1013.

Additional file S2: The definition of VAP

According to the 2012 ventilator-associated pneumonia (VAP) diagnostic criteria proposed by the US Centers for Disease Control/National Healthcare Safety Network^1,2^, patients with mechanical ventilation for >48 hours have worsened oxygenation when the condition is relatively stable, and thus the oxygen concentration or PEEP need to be increased. VAP needs to be suspected when: 1) New or worsening pulmonary symptoms or signs occur; 2) Temperature rises >38 °C or falls <36 °C, or white blood cell count ≥12,000 cells/mm^3^ or ≤4,000 cells/mm^3^; and/or 3) Chest radiographs show the presence of new or progressive infiltration. VAP is confirmed by positive culture from one of the following specimens and meets the quantitative or semi-quantitative thresholds as outlined in this protocol, without requirement for purulent respiratory secretions: 1) Endotracheal aspirate, ≥10^5^ CFU/ml or corresponding semi-quantitative result; 2) Bronchoalveolar lavage, ≥10^4^ CFU/ml or corresponding semi-quantitative result; 3) Lung tissue, ≥10^4^ CFU/g or corresponding semi-quantitative result; and/or 4) Protected-specimen brush, ≥10^3^ CFU/ml or corresponding semi-quantitative result.

**References:**

1. Magill SS, Klompas M, Balk R, Burns SM, Deutschman CS, Diekema D, et al. Developing a new, national approach to surveillance for ventilator-associated events. Crit Care Med 2013;41:2467-2475.

2. Klompas M. Complications of mechanical ventilation-The CDC’s new surveillance paradigm. N Engl J Med 2013;368:1472-1475.

Table S1. Hemodynamic status and Laboratory examinations of study patients at randomization

| Patient Characteristics | Total (n=241) | RFEM (n=120) | SSD (n=121) | *p* |
| --- | --- | --- | --- | --- |
| Hemodynamic status |  |  |  |  |
| Systolic blood pressure (mmHg) | 123±24 | 125±23 | 121±24 | 0.316 |
| Diastolic blood pressure (mmHg) | 69±14 | 71±13 | 67±15 | 0.220 |
| Heart rate (beats/min) | 104±25 | 100±23 | 108±27 | 0.108 |
| Lactate (mmol/L) | 1.6 (1.1-2.3) | 1.6 (1.0-2.1) | 1.6 (1.2-2.4) | 0.646 |
| Laboratory examinations |  |  |  |  |
| White blood cell (×10^9^/L) | 9.46±6.60 | 9.84±6.44 | 9.04±6.82 | 0.557 |
| Neutrophil (×10^9^/L) | 8.37±5.85 | 8.59±5.56 | 8.13±6.23 | 0.705 |
| Hemoglobin (g/L) | 112.74±26.69 | 110.92±24.55 | 114.80±29.07 | 0.480 |
| Platelet count (×10^9^/L) | 165.92±99.08 | 173.84±105.49 | 156.93±91.61 | 0.407 |
| Albumin (g/L) | 27.21±5.81 | 27.6±5.4 | 26.8±6.3 | 0.510 |
| Alanine aminotransferase (U/L) | 34.0 (21.0-65.0) | 30.5 (17.0-64.5) | 36.0 (22.0-64.5) | 0.589 |
| Aspartate aminotransferase (U/L) | 47.0 (30.0-109.0) | 45.5 (25.5-93.0) | 57.0 (32.0-144.5) | 0.245 |
| Total-bilirubin (umol/L) | 11.6 (6.9-17.0) | 11.6 (7.2-14.8) | 11.9 (6.6-20.2) | 0.982 |
| Direct-bilirubin (umol/L) | 5.1 (3.0-8.5) | 4.9 (3.0-7.8) | 5.4 (3.0-10.0) | 0.631 |
| Urea nitrogen (mmol/L) | 6.9 (5.2-12.1) | 7.2 (5.7-12.5) | 6.2 (4.9-11.5) | 0.178 |
| Creatinine (umol/L) | 80.0 (52.2-119.4) | 84.3 (54.8-111.7) | 65.5 (50.2-128.9) | 0.660 |
| Troponin I (ng/ml) | 0.03 (0.00-0.14) | 0.02 (0.00-0.12) | 0.03 (0.00-0.17) | 0.440 |
| N terminal pro type B natriuretic peptide (×10^3^pg/ml) | 1.05 (0.64-1.66) | 1.15 (0.73-1.66) | 1.02 (0.43-1.89) | 0.301 |
| C-reactive protein (mg/dL) | 13.9 (10.8-18.7) | 14.6 (11.5-27.7) | 13.8 (8.9-17.9) | 0.160 |
| Procalcitonin (ng/ml) | 2.90 (0.68-11.1) | 4.37 (0.87-11.23) | 1.51 (0.39-11.15) | 0.173 |

Table S2.Tracheal intubation due to respiratory failure

| Respiratory failure, n (%) | Total (n=174) | RFEM (n=83) | SSD (n=91) | *p* |
| --- | --- | --- | --- | --- |
| Pneumonia | 110 (63.22) | 50 (60.24) | 60 (65.93) | 0.437 |
| Bacterial | 16 (14.55) | 7 (14.00) | 9 (15.00) | 0.740 |
| Fungal | 8 (7.27) | 3 (6.00) | 5 (8.33) | 0.723 |
| Viral | 28 (25.45) | 14 (28.00) | 14 (23.33) | 0.790 |
| Atypical pathogens | 3 (2.73) | 2 (4.00) | 1 (1.67) | 0.606 |
| mixed infection | 32 (29.09) | 14 (28.00) | 18 (30.00) | 0.620 |
| undefined | 23 (20.91) | 10 (20.00) | 13 (21.67) | 0.663 |
| AE-COPD | 29 (16.67) | 17 (20.48) | 12 (13.19) | 0.197 |
| Pulmonary fibrosis | 19 (10.92) | 7 (8.43) | 12 (13.19) | 0.315 |
| Bronchiectasis with secondary infection | 5 (2.87) | 3 (3.61) | 2 (2.20) | 0.670 |
| Asthma | 4 (2.30) | 2 (2.41) | 2 (2.20) | 1.000 |
| Trauma | 3 (1.72) | 1 (1.20) | 2 (2.20) | 1.000 |
| Pulmonary tuberculosis | 2 (1.15) | 1 (1.20) | 1 (1.10) | 1.000 |
| Diffuse alveolar hemorrhage | 1 (0.57) | 1 (1.20) | 0 | 0.477 |
| Pulmonary embolism | 1 (0.57) | 1 (1.20) | 0 | 0.477 |

Definition of abbreviations: AECOPD=acute exacerbation of chronic obstructive pulmonary disease.

Table S3. Risk factors for VAP during the study period

| Risk factors | RFEM (n=120) | SSD (n=121) | P value |
| --- | --- | --- | --- |
| Prior antibiotic therapy, n(%) | 105 (87.50) | 100 (82.64) | 0.290 |
| Minimize sedation, n(%) | 92 (76.67) | 95 (78.51) | 0.731 |
| Elevating head of bed to 30 degree, n(%) | 84 (70.00) | 76 (62.81) | 0.237 |
| Maintain the cuff pressure at 25-30 cmH_2_O, n(%) | 102 (85.00) | 106 (87.60) | 0.557 |
| Daily oral hygiene, n(%) | 120 (100.00) | 121 (100.00) | 1.000 |
| Prevention of gastric overdistension, n(%) | 62 (51.67) | 66 (54.55) | 0.654 |
| Enteral nutrition, n(%) | 112 (93.33) | 110 (90.91) | 0.485 |

Table S4. Microorganisms diagnosis of VAP in patients

| Pathogens | RFEM (n=11) | SSD (n=13) | P value |
| --- | --- | --- | --- |
| Gram-negative bacteria, n (%) |  |  |  |
| Acinetobacter baumannii | 7 (63.64) | 8 (61.54) | 1.000 |
| Pseudomonas aeruginosa | 3 (27.27) | 3 (23.08) | 1.000 |
| Klebsiella pneumoniae | 0 | 1 (7.69) | 1.000 |
| Escherichia coli | 0 | 1(7.69) | 1.000 |
| Gram-positive bacteria, n (%) |  |  |  |
| Streptococcus constellatus | 1 (9.09) | 0 | 0.458 |

Table S5. Changes of vital signs during process of rapid-flow expulsion maneuver

|  | Pre-maneuver | Worst value during maneuver | Z value | P value |
| --- | --- | --- | --- | --- |
| Heart rate, beats/min | 91 (80-103) | 98 (87-111) | -20.267 | <0.001 |
| Respiratory rate, times/min | 20 (15-25) | 25 (19-30) | -18.120 | <0.001 |
| Systolic blood pressure, mmHg | 121 (109-132) | 130 (115-143) | -16.699 | <0.001 |
| Diastolic blood pressure, mmHg | 63 (58-70) | 68 (61-74) | -12.769 | <0.001 |
| Pulse oxygen saturation, % | 97 (95-98) | 96 (95-99) | -1.730 | 0.084 |
